# Age Differences in the Effectiveness of Telehealth Mindfulness-Based Interventions for Chronic Pain

**DOI:** 10.1093/geroni/igaf122.2127

**Published:** 2025-12-31

**Authors:** Kushang Patel

**Affiliations:** University of Washington, Seattle, Washington, United States

## Abstract

Mindfulness-based interventions (MBIs) are evidence-based treatments for managing chronic pain in adults. However, few studies have assessed heterogeneity in the effectiveness of remotely delivered MBIs by age. Given the high burden of pain among older adults and the potential scalability of telehealth MBIs, we explored potential age differences in the effectiveness of two 8-week telehealth MBIs. The primary outcome was pain interference as measured by the Brief Pain Inventory scale (higher scores are worse) collected at baseline, 10 weeks, 6 months, and 12 months. A total of 811 Veterans with moderate-to-severe chronic pain were randomized to either synchronous group-based MBI (n = 270), asynchronous self-paced MBI (n = 271), or usual care (n = 270). The numbers of older participants (age ≥65 years) by treatment assignment were 59 (21.9%) in group-based MBI, 71 (26.2%) in self-paced MBI, and 58 (21.5%) in usual care. Adjusting for gender, study site, and baseline pain interference and self-efficacy, older participants in self-paced MBI reported significantly less pain interference compared with those in usual care at 10 weeks (mean difference=-0.7 [95% CI: -1.3, -0.1]), but there were no significant between-group differences at 6 and 12 months. In contrast, there were no significant differences in pain interference between older participants in group-based MBI versus usual care at any post-treatment timepoint. Among younger participants (<65 years old), both MBIs significantly decreased pain interference at each post-treatment timepoint compared with usual care. Findings suggest that self-paced MBI can improve pain interference among older Veterans. Larger trials of telehealth MBIs in older adults are needed.

